# Coincidental discovery of HIV and pregnancy positive status in primary healthcare facilities

**DOI:** 10.4102/curationis.v47i1.2518

**Published:** 2024-06-28

**Authors:** Morongwa R. Sekele, Mygirl P. Lowane, Mathildah Mokgatle

**Affiliations:** 1 Department of Public Health, Faculty of Healthcare Sciences, Sefako Makgatho Healthcare Sciences University, Ga-Rankuwa, South Africa

**Keywords:** acceptance and transition, grief and bereavement, HIV and pregnancy, mother to child transmission, psychological distress

## Abstract

**Background:**

HIV-positive and pregnancy diagnosis is a traumatic, shocking, and distressing experience for women. Adoption of routine HIV counselling and testing in the antenatal programme aimed to increase the uptake and the early diagnosis of HIV among pregnant women to prevent maternal HIV transmission to unborn babies and neonates.

**Objectives:**

The study aimed to explore the psychological reaction of women coincidentally discovering their HIV-positive status and pregnancy while seeking medical care in primary healthcare facilities in the Tshwane district.

**Method:**

Descriptive phenomenology involving a semi-structured in-depth interview was used to collect data. The sample was purposively selected. Twenty-eight women participated in the research project. Face-to-face in-depth audio recorded interviews were used to gain a full understanding of the experiences and feelings of the participants.

**Results:**

Reason for the uptake of pregnancy and HIV testing, reactions upon discovering HIV and pregnancy-positive status, emotions arising from the pregnancy and HIV-positive diagnosis, understanding HIV infection in pregnancy, and transitions to acceptance and coping with the HIV-positive diagnosis were themes that emerged from this study.

**Conclusion:**

It is crucial that responsible healthcare workers consider this psychological imbalance during their offering of antenatal and postnatal care services so that the pregnant women living with HIV can accept and cope with the situation.

**Contribution:**

This study accounts to support other studies that offer intense counselling for women coincidentally discovering their positive HIV status and pregnancy. It is important to remedy the acceptance of the situation and to promote HIV prevention and family planning for women of childbearing age.

## Introduction

Because of the alarming increase in the number of HIV-infected women, in 2013 South Africa responded to the recommendation of the United Nations Programme on HIV/AIDS (UNAIDS) and the World Health Organization (WHO) to implement routine HIV testing of all pregnant women in an attempt to increase the HIV testing rates and the prevention of the mother to child transmission (PMTCT) of HIV in all healthcare facilities (Landes et al. 2021). Pregnancy was considered to be a gateway for HIV diagnosis in women of reproductive age (Ansah et al. 2021; Feyissa et al. 2019). In South Africa, pregnant women are often encouraged to test their HIV status during their routine visits to the antenatal clinic and those diagnosed with HIV are often motivated to take antiretroviral therapy (ART) on the same day as their HIV diagnosis to prevent HIV transmission to their unborn infant during their pregnancy (Feyissa et al. 2019; WHO 2021).

HIV testing and counselling (HTC) are critical services provided to pregnant women (Adu et al. 2023; Oshosen et al. 2021). The testing was found to be essential for identifying HIV-infected women who needed to start antiretroviral treatment (Woldesenbet et al. 2021). Since the advent of large-scale antiretroviral treatment, HIV testing practices among pregnant women during antenatal services have changed dramatically (Ansah et al. 2021). The PMTCT in South Africa has brought about routine HIV testing for pregnant women attending antenatal clinics (Kim et al. 2018). The 2013 WHO guidelines recommend that all pregnant and breastfeeding women found to be HIV-positive during pregnancy should start ART and continue with lifelong treatment (Landes et al. 2021). These guidelines support the implementation of the Global Plan for the elimination of new HIV infections that may occur among infants and for keeping their mothers alive (Madiba & Putsoane 2020; Woldesenbet et al. 2021).

New maternal HIV infections among pregnant women contribute significantly to the mother to child transmission of HIV (MTCT), and in 2019 new maternal HIV infections during pregnancy were reported to be the second major cause of perinatal HIV transmission globally (Chilaka & Konje 2021; Harris & Yudin 2020). New maternal HIV infections in South Africa accounted for an estimated 26% of the vertically transmitted early infant HIV infections (Woldesenbet et al. 2021). The adoption of routine HIV counselling and testing (HCT) in the antenatal programme has increased the uptake of HIV testing services and the early diagnosis of HIV among pregnant women (Aba Abraham & Clow 2022). It is indicated that there are still pregnant women presenting late to antenatal clinics and the risk of MTCT is significantly higher with them than with mothers who receive ART in the early stage of pregnancy (Alhassan et al. 2022). A high proportion of pregnant women in sub-Saharan Africa (SSA) discovered their HIV-positive status for the first time during antenatal testing, screening and counselling (Ansah et al. 2021). Pregnant women newly diagnosed with HIV are faced with the decision to commit to lifelong ART, and this decision is also often based on the health of their infants (Feyissa et al. 2019)

Despite the benefit of being enrolled in the PMTCT of the HIV Programme, receiving an HIV-positive diagnosis during their pregnancy is a traumatic, shocking, and distressing experience for women (Kim et al. 2018). Pregnant women diagnosed with HIV often face the dual burden of having to deal with their pregnancy and the fear of transmitting HIV to their babies (Ansah et al. 2021). The mother not only fears infecting her infant but is also concerned over her own health and doubts whether she will be able to provide for the baby (Kim et al. 2018). Among other the traumatic aspects of being told about their HIV-positive status soon after being diagnosed with pregnancy, the mothers are also faced with the issue of the disclosure of their HIV status and the need to take the decision to start lifelong ART.

Pregnant women who are diagnosed with HIV experience numerous additional stressors such as financial problems, lack of support from their partners, and concern for the physical well-being of their children, and these problems increase the risk of postpartum depression among HIV-positive women (Moseholm et al. 2022). Research suggests that an HIV diagnosis can act as a traumatic stressor and is associated with mental distress and trauma (Ayano, Duko & Bedaso 2020; Yemeke, et al. 2020). Despite all these challenges, some women diagnosed with HIV during pregnancy are more concerned about the well-being of their unborn children, and this concern motivates them to cope with their HIV-positive status and to adhere to ART (Drigo et al. 2020; Ebonwu et al. 2018).

Social and contextual factors influence women’s reaction to their HIV diagnosis during their pregnancy (Kim et al. 2018). The stigma that communities attach to HIV as well as culturally entrenched gender roles give rise to and still perpetuate the occlusion of the disease in a veil of silence and fear. These factors contribute to women’s psychological distress upon discovering their status (Kim et al. 2018). An HIV-positive diagnosis can be a life-altering event with detrimental emotional and psychological effects (Payán et al. 2019). A study conducted in Zambia also found that women diagnosed during pregnancy were more likely to experience psychological distress than pregnant women who had prior knowledge of their HIV-positive status (Kim et al. 2018; Sianchapa, Katowa-Mukwato & Ngoma 2024). They experienced worse loss of interest in life, feelings of worthlessness, suicidal ideation and anxiety, and were more inclined to consider terminating their pregnancy (Kim et al. 2018). They were inclined to express anger and confusion about the impact of the HIV diagnosis and recognised the fact that they had lost something of value (Burns et al. 2024).

HIV is one of the major public health problems, especially in developing countries, accounting for approximately 12% of maternal deaths during pregnancy and 2% during postpartum (Arias-Colmenero et al. 2020; Kalungwe et al. 2022). Africa is the most affected region worldwide, where most of the population including pregnant women is currently being treated with antiretroviral therapy (Arias-Colmenero et al. 2020). A similar situation arises in South Africa, where pregnant women are diagnosed with HIV at antenatal clinics and are simultaneously placed on antiretroviral treatment (ART) (Drigo et al. 2020). Antiretroviral therapy was found to be the most effective way to reduce HIV-related maternal mortality and perinatal transmission of HIV (Moseholm et al. 2022). HIV-positive pregnant women among other worries face poor adherence to ART, as a result, this affects the progress in the fight against maternal mortality (Kalungwe et al. 2022). Despite these benefits, pregnant women must also cope with stressors that are affecting their health (Moseholm et al. 2022). They also have to deal with undergoing seropositive stages. These stages include the stage of diagnosis, shock; negative adaptation (denial), or positive adaptation (acceptance) (Arias-Colmenero et al. 2020). An HIV diagnosis together with the initiation of ART has an emotional impact that may influence how the women cope with their pregnancy and their adherence to a treatment plan.

Although studies have been conducted to investigate women’s views of the acceptability of the antenatal HIV test in pregnancy and the giving of pre-test information (Pius et al. 2023; Santos Melo et al. 2021), there are limited studies that explore the reactions of women receiving HIV and pregnancy-positive results simultaneously. Thus, the study aimed to explore the reactions of women coincidentally discovering their HIV-positive status and pregnancy while seeking medical care in primary healthcare facilities in the Tshwane district, Gauteng Province, South Africa.

## Research methods and design

### Study setting and design

The study was conducted in Tshwane Sub-District Region 5, which is a rural area of the Gauteng Province of South Africa. The sub-district has three public healthcare clinics and one municipal healthcare clinic and one hospital, all of which provide comprehensive antenatal services and other HIV-related services, including PMTCT services. The study was conducted in two public healthcare clinics and in one municipal healthcare facility. The three healthcare facilities were purposely selected because of the large number of pregnant women accessing antenatal services in those facilities.

The population of the study comprised pregnant women who are HIV-positive and are enrolled in the PMTCT Programme in Tshwane sub-district Region 5. HIV-positive pregnant women were recruited from the clinics. Purposive sampling was employed to select a sample of women who met the criteria for inclusion in the study. Women who were diagnosed with HIV and pregnancy simultaneously and who were still pregnant at the time of data collection were considered for inclusion criteria. However, women who knew their pregnancy status when diagnosed with HIV and vice versa were excluded from this study.

To ensure that their lived experiences were captured appropriately, the study adopted a methodology relying on Husserl’s descriptive qualitative phenomenology. A qualitative methodological approach is fundamental to a realisation of the personal life journey of human beings and the description of their experiences in depth (Bowling 2014). Phenomenology attempts to understand the holistic nature of phenomena as people experience them (Lingen-Stallard, Furber & Lavender 2016). Thus, this study is a collection of descriptions of meanings for individuals, and their lived experiences of phenomena (Bowling 2014). Furthermore, reliance on Husserl’s phenomenology led to the attempt to penetrate deeper and deeper into the reality of the experience of being diagnosed with HIV during pregnancy, and to the exploration of the perceptions, understandings, and feelings of the participants about the phenomenon encountered (Sloan & Bowe 2014).

### Sample size

The study presupposed that the phenomenon of HIV testing would be a sensitive, personal, life experience. The size of the sample could thus have been influenced by the amount of in-depth data to be drawn from the individual participants’ experiences of being given their HIV-positive diagnoses during their antenatal visits. The sample size in this study consisted of 28 women. Data saturation was observed when no new information was coming up from subsequent interviews after the 25th participant. However, the interview continued with three more participants (one in each setting) to confirm if there was no new information coming up.

### Access and recruitment

The healthcare managers at the selected facilities were approached asking permission to utilise their facility to conduct the interviews with the participants. As the issue of HIV is very sensitive by nature, the investigator liaised with the midwives responsible for HCT in the antenatal section and asked them to inform eligible women about the study. Those interested in the study were provided with options to self-refer, to allow the midwife to give the investigator their contact details, or to be directly introduced to the investigator immediately to be fully informed about the study and to grant their informed consent if they decided to participate.

### Data collection

Face-to-face in-depth interviews were used as a method of gaining a full understanding of the experiences and feelings of the participants. A self-interview guide was developed based on the literature reviewed of the same study context, and the objective of the study was also considered. The in-depth interview guide was developed in English and then translated into Setswana the local language used on that setting by the first author who is conversant with that language. The first author then conducted all interviews in Setswana language to allow the participants to freely express themselves in their own language. Open-ended questions, follow-up questions, and probing were employed to facilitate conversation with each participant, and to get the meaning of what the participants raised. The data gathered in this way were supported by field notes. With their consent a digital audio recorder was also used to record the contents of the interview. Some of the questions that were asked during the interview included: How did you find out that you are HIV-positive? How did you find out that you are pregnant? After you were told that you are HIV-positive how did you feel? Who did you tell about your HIV-positive status? Now that you know that you are HIV-positive, how do you feel about your pregnancy? What are you fears regarding your HIV status and pregnancy? What has your relationship with your partner been like since you told him about your HIV status and pregnancy? Tell me about your experiences of being on treatment for HIV and your pregnancy.

An arrangement was made that the interviews would take place during the participants’ ANC visits in one of the consulting room provided by the facility manager to ensure confidentiality and privacy. The mothers were recruited from the antenatal care (ANC) clinic in each facility. Women meeting the inclusion criteria were considered for the study. The midwife in the ANC section identified those who met the inclusion criteria and referred them to the principal investigator (first author) at the allocated consulting room after they had completed their routine ANC consultations. Arrangements for the interview were made after the investigator presented the purpose of the study. For those who opted to be interviewed on the same day, the interview commenced immediately after obtaining informed consent. Appointments were made with those who deferred the interviews. An interview lasted for approximately 45–60 min. In cases where the woman became upset during the interview, an option to terminate was given and referral to the resident social workers was offered. However, no participant terminated the interview or decided to withdraw from the study. Data collection occurred over a period of 9 months from April 2017 to December 2017, respectively.

### Trustworthiness

The four principles described by Lincoln and Guba in Nassaji (2020), namely credibility, dependability, transferability, and transferability, were abided by to ensure trustworthiness. The credibility of the study was ensured by awarding each subject an opportunity to refuse to participate or to withdraw at any stage of the study. This was done to ensure that the data were collected only from people who were genuinely willing to participate and who would give honest responses. Dependability was ensured through checking of the findings with the participants and keeping an audit trail for use, should an independent review be required, whereas conformability was ensured through the bracketing process to ensure that the data generated were not skewed by the imposition of the investigator’s opinions (Nassaji 2020), Lastly, transferability was observed by ensuring that the results of the study were accurately collected, so that they could be applied to similar situations that might occur in the future.

### Analysis

The analysis was led by the main author, aided by input from the other authors. The data derived from the interviews were transcribed verbatim and translated from Setswana to English. The investigator (first author) checked the translation of the interviews for accuracy. Individual transcripts were read again and again to get a sense of the contents and context of the data collected (Denzin & Lincoln 1998; De Vos et al. 2002). Recorded interviews were listened to concurrently with the field notes to identify other aspects such as non-verbal gestures, pauses, and laughter, and to verify if such indicators were included in the transcripts. The data were analysed through the utilisation of qualitative data analysis methodology and open coding. Open coding refers to the process of basic descriptive coding of the content of narrative materials (Renjith et al. 2021). Initial coding was performed manually to identify the central emergent themes. Thematic content was used to identify common themes from each transcript; thereafter a code was developed. Central themes emerged through the process of reading, re-reading, and coding the transcripts, and a written description and interpretation of the data was carried out. Quotations representing the themes identified were selected and used to illustrate the findings. All authors assisted in developing the code list to ensure the accuracy of the analysis and interpretation of the data. NVIVO version 10 software was used to manage and analyse the data that emerged from the transcripts.

### Ethical considerations

Ethical clearance for this study was obtained from the Research and Ethics Committee of Sefako Makgatho Health Sciences University (SMUREC/H/163/2016:PG) and from the Department of Health in Tshwane sub-district. The safety and well-being of the participants was a primary concern in this study, and they were treated with respect and dignity. Informed consent was obtained from the participants, and they were told that their participation was voluntary. Only participants who signed the consent form participated in the study. Individual interviews were conducted in a private consulting room to ensure privacy. Pseudonyms were used during the interview to label the transcripts and to present the quotations to ensure confidentiality and anonymity of the participants.

## Results

A sample of 28 HIV-positive pregnant women participated in the study. However, gaps were identified when analysing the sociodemographic data. Where ‘*n*’ was found to be less than 28 as illustrated in Table 1, it was because of non-response to some sociodemographic questions. Twelve participants were from Clinic A, seven were from Clinic B, and nine were from the municipality clinic. Their ages ranged from 18 to 41 years old, with a mean age of 27 years. At the time of the interviews, all the participants were on ART. Twenty-seven participants were single but in some form of relationship and one was married. Most of them had attended school; 22 of them up to the secondary level. Twenty-one of the participants were unemployed, whereas, six were employed. The participants’ characteristics are illustrated in Table 1.

**TABLE 1:** Demographic characteristics of the participants in the study.

Variable	Subcategory	Frequency (*n*)
Age (years) category (*N* = 28)	18–20	4
	21–25	7
	26–30	7
	31–35	4
	35 +	6
Marital status (*N* = 28)	Single	27
	Married	1
Living arrangements (*N* = 26)	My partner and children	14
	My children only	1
	My children and my parents	11
Employment status (*N* = 27)	Employed	6
	Unemployed	21
Level of education (*N* = 28)	Primary school	2
	High school	25
	Tertiary education	1
Religion (*N* = 27)	Christian	27

### Participants characteristics

The themes and sub-themes that emerged from the data focused on the participants’ reactions after receiving a HIV-positive and pregnancy diagnosis while seeking medical consultation.

### Reasons for the uptake of pregnancy and HIV testing

A common reason that was given for taking the pregnancy test was that the participant had not been feeling well and so decided to go to the clinic. Some participants indicated that they had not even been aware that the sickness might be pregnancy-related, whereas others said that because of the state of their ill-health they decided to go to the clinic for HIV testing but also got tested for pregnancy. Below are a few excerpts to illustrate this:

‘I had a back pain, I couldn’t eat, I did not have an appetite and then I decided to go to the clinic. When I got there, they tested me for pregnancy and found out that the test is positive [*pregnancy*] then sent for HIV testing.’ (Leah, 28 years old)

‘I went to the clinic because I have missed my menstrual period. A pregnancy test was done and was positive. Then I was sent to the counselling room for HIV testing and found out that the test [*HIV*] are also positive too.’ (Carlota, 22 years old)

Some participants spoke of having other reasons for taking the HIV tests. Some of them had been motivated to accept the HIV tests simply because they had felt they were vulnerable to contracting HIV:

‘My partner was cheating on me, so I decided to do an HIV test, only to find out I am positive. Pregnancy test was also done and was positive also …’ (Lebogang, 24 years old)

### Reactions from discovering the HIV and pregnancy positive status

The participants had mixed reactions to the HIV and pregnancy diagnoses. Some participants felt disappointed, while others were shocked or angry or reacted with disbelief. Most of the participants found it difficult to come to terms with being HIV-positive and pregnant at the same time. Three sub-themes emerged from this theme.

#### Experience of shock and disbelief

Most participants described the impacts of being HIV-positive and pregnant as traumatic. Almost all of them reported that they were shocked and found it hard to believe in their HIV and pregnancy status. A participant reported that it was hard for her to believe as all the previous HIV tests she had taken had come out negative:

‘I was shocked because I have been testing all along and I always tested negative. Now I really do not know what went wrong.’ (Thandi, 18 years old)

Some participants had not believed their HIV results to be true:

‘I cried when they broke the news of HIV. I kept on asking myself how possible because I am not cheating … yaaa … for pregnancy I might understand but this [*HIV*] no, no, no.’ (Pulane, 27 years old)

Disbelief was identified to be common among the women who had been HIV-negative with their previous pregnancies:

‘When the nurse informed me that I am HIV-positive, I did not believe it, because I never thought that I was much at risk of getting HIV. I am married and faithful to my husband and have three kids already, so how come with this one …’ (Linkie, 26 years old)

Some participants reacted with expressions of denial and reported mixed feelings after learning of their diagnosis and psychologically blocked the fact that they were HIV-positive:

‘I did nothing. I felt like the world has come to an end. Actually, I don’t know how I feel. I had mixed emotions. It can’t be true.’ (Thandi, 18 years old)

#### Worry about vertical transmission of HIV to the unborn child and the effect of the illness

The worry that prevailed among some participants included fearing transmission of the HIV virus to their unborn child. HIV transmission of the virus to the unborn child was found to be the main concern of some of the participants. One of them cried when she said:

‘The fact that I’m HIV-positive, I really do not know whether the child will be born without HIV. I think about such things.’ (Linkie, 26 years old)

Even though she was on the PMTCT programme, one participant expressed her worry and fear that she might still infect her unborn child:

‘My fear is that I wish that the virus that I am having must not affect the child and that this child should be the last one because this proves to me that this thing is dangerous, and I’m HIV-positive taking treatment, I’m scared that the baby might come out HIV-positive even though I’m taking ARVs, and I’m still young to be having stress. I’m fine. I’m just scared for the unborn baby.’ (Caroline, 29 years old)

All the participants had received pre-counselling prior to being tested for HIV during antenatal care. Despite the information they had received before and after testing, some participants were worried about the potential outcome for their infants, even though they were taking precautions to prevent MTCT. Worrying about MTCT for the children went beyond their survival because of the HIV infection. The participants were greatly concerned for their future and their children’s future. Mainly, they were concerned about the lack of care for the child if they become critically ill or succumbed to the illness:

‘What if I die, who is going to take care of my child? What will happen to my child if she finds out I’m HIV-positive? Those were the things that came to my mind.’ (Mathapelo, 41 years old)

### Emotions arising from the pregnancy and positive HIV diagnosis

To some participants being pregnant and HIV-positive at the same time affected their psychological and emotional well-being. Describing their reactions to finding out about their HIV and pregnancy diagnosis, most participants said they were extremely hurt, sad, depressed and stressed, and felt hopeless:

‘I was a bit stressed. I was worried.’ (Linkie, 26 years old)

‘For the first time I hear that I was so hurt. My heart was sore but I told myself that there is nothing I can do, but even now I am still hurt. Eish! I was thinking a lot. I was not sleeping.’ (Lebogang, 20 years old)

Some of them felt that their lives had completely changed. Some expressed confusion after the diagnosis:

‘I did nothing, I felt like the world has come to an end. Actually I don’t know how I was feeling, I had mixed emotions.’ (Thandi, 18 years old)

A deep sense of sadness was experienced by some of the participants. Some expressed their emotions by reporting that they were depressed, meaning heartbroken, and others expressed their depression by constant crying:

‘I cried when they broke the news of HIV. I kept on asking myself how possible because I am not cheating. For pregnancy, I might understand but this [*HIV*] no, no, no.’ (Pulane, 27 years old)

Despite the depressing situation, two participants reported that they had experienced emotional support from their family members who showed compassion towards them:

‘I felt saddened and depressed before. But I spoke to my mother, and she said to me I must not worry as long as I adhere to treatment. I disclosed it to my mother because she was the one, I trust …’ (Maria, 19 years old)

‘I was heartbroken. I felt extremely depressed. When I got home, I told my husband about my pregnancy and HIV status.’ (Above, 36 years old,)

### Understanding HIV infection in general and during pregnancy

Some participants indicated that they had had no idea about the HIV infection. They had learnt about it when they were enrolled in the PMTCT programme:

‘To be honest with you, I know nothing. I started knowing that I am HIV-positive when they tested me during my pregnancy. It’s for the first time.’ (Caroline, 29 years old)

‘I don’t [*know about HIV*] because I never thought anything about it. They only tested me, and I was told I have HIV and they gave me pills.’ (Jane, 38 years old)

‘I don’t know anything about HIV because I only found out that I am HIV-positive when I came for testing right now during my pregnancy. During my first pregnancy I was not HIV positive. That is why I’m saying I have no information about HIV. I only found out now when I was testing.’ (Lerato, 24 years old)

The participants showed varying degrees of understanding of the HIV disease. Some knew very little while others understood it mostly through the lens of sexual transmission:

‘Actually, before testing for HIV, I had a lot of information with regards to HIV because most of my family members have HIV, so I had some information about it.’ (Mathapelo, 41 years old)

‘What I know is that if you don’t take treatment your body system is going to be weak, you will start getting sick, and when you become pregnant you must take treatment so that you can deliver a healthy child. You also have to adhere to treatment.’ (Brenda, 23 years old)

#### Transitions to acceptance and coping with the HIV-positive diagnosis

The sequence of feelings participants experienced as they adjusted to living with HIV in pregnancy started with shock, then disbelief, and finally acceptance of the situation. At first most of the participants reacted with shock and were saddened by the fact that they had transited from being HIV-negative to HIV-positive. However, some thought that accepting and coping with the illness was better:

‘I felt sad, but I accepted that I’m HIV-positive.’ (Sharon, 26 years old)

Mapule, 35 years old, thought before taking the test that she might be faced with serious mental challenges if she was found to be HIV positive. In contrast, she accepted her status without any difficulties. She highlighted the fact that she did not have a problem despite the fact that she once told herself that she would commit suicide if she could get an HIV infection. She went on to indicate that ‘I accepted and took a decision that I will take treatment for the sake of my children’.

Most of the participants had tried to embrace feelings of acceptance soon after they learnt about their HIV and pregnancy status. At some point, they became motivated to accept and cope with the illness by learning from their family members who were already living with HIV:

‘I didn’t feel bad because at home I’m staying with people who have HIV. I accepted. I told myself that I’m now sick and I have to take my treatment. I made sure that I inform them at home that I’m now on ARVs. They said to me I must make sure that I adhere to treatment, and I will be fine. Just look at my body. It doesn’t show that I’m taking ARVs. Most people don’t believe that I’m taking ARVs.’ (Mathapelo, 41 years old)

Two participants’ response was not showing empathy:

‘I accepted because when I checked I’m not the only one who has HIV. Most people are HIV-positive.’ (Lebo, 26 years old)

‘No, I have accepted that I am HIV-positive, and I am going to live with it so there is no problem. I am fine. I am taking treatment to make sure that the children don’t get infected. That’s the only thing that I am doing.’ (Mpumi, 22 years old)

## Discussion

The study explored the reactions of women who discovered their pregnancy and HIV status simultaneously. Women visit healthcare facilities to access various services and this study revealed that most of the participants had been going for general consultations because of not feeling well, while some reported that they have some gynaecological issues. Some of the participants had been attending the clinics only to take HIV tests because their partners reported infidelity when they also discovered their pregnancy.

The participants in this study spoke openly about their reactions to discovering the positive results of the HIV and pregnancy tests. They reported experiencing a state of shock and disbelief. This reaction is reported in various other studies as well (Akinsolu et al. 2023; Arias-Colmenero et al. 2022; Putsoane & Madiba 2021). Moreover, all of those who participated in the study were worried about their unborn babies. Vertical transmission to the unborn baby happens mostly if the mother’s viral load is high (Chilaka & Konje 2021). The study revealed that the participants were not even aware of their HIV status; this suggests that their viral load was higher because they were not on ART at the time of the diagnosis with HIV and pregnancy.

The literature indicates that the viral load becomes high when the person becomes infected (Chilaka & Konje 2021; Moodley et al. 2021), hence early enrolment in the PMTCT programme is important to reduce the vertical transmission of HIV to unborn babies.

The participants in this study suffered emotionally because of the dual predicaments of being pregnant and HIV-positive at the same time. Previous studies have also revealed that women in that situation are prone to depression and engage in suicidal ideation (Legazpi et al. 2022; Rodriguez et al. 2018). Special attention should be given to the care and support of HIV-positive and pregnant women because, according to the literature, emotional distress may result in poor adherence to ART in the general population (De Los Rios et al. 2021; Van Wyk & Kagee 2023). This implies that emotional distress among pregnant women who are diagnosed with HIV may lead to other severe mental health conditions (Waldron et al. 2021). Mental health problems in pregnancy may also affect the condition of the unborn baby (Van Den Bergh et al. 2020). The literature indicates that most women with puerperium psychosis suffered mental health challenges during pregnancy (Chilaka & Muriithi 2021).

The study also reveals that some of the participants were knowledgeable about HIV, but others reported that they were lacking in knowledge regarding HIV and MTCT. Various studies have reported the same findings (Deynu & Nutor 2023; Mudji et al. 2023; Yeshaneh et al. 2023), and a study conducted in Tanzania confirmed that a low level of knowledge on MTCT is common among *nil para gravida* women (Nsibande et al. 2022). These findings suggest that there is a need to expand HIV educative programmes, especially in disadvantaged rural communities. Health education, HCT uptake should also be strengthened so that people can be informed about HIV. This strategy may help to reduce new HIV infections in future. Moreover, the mother’s understanding of HIV infection during antenatal and postnatal care might improve her adherence to the PMTCT programme and even alleviate her fears and worries.

The participants in this study expressed acceptance of and the ability to transition to living with HIV. They also indicated how they feel and how are they going to live, moving forward. Some had gone to the extent of disclosing their status to their partners and to their family members. These two phenomena of acceptance and transformation reflect a model of grief and bereavement by Kübler-Ross (Gerhardt & Puchkov 2023; Payán et al. 2019). It is evident that the women need to be supported emotionally and psychologically in response to the fact that they experience loss on discovering their HIV and pregnancy status without being prepared for this (Madiba 2021). Pregnant HIV-positive women experience two predicaments in that they are HIV-positive, yet they are responsible for bringing a healthy new life into being. Therefore, special care and support from healthcare providers responsible for antenatal and postnatal care services and support from the partners and family members are needed to promote a relatively healthy life for both the mother and the baby.

### Limitations

The findings of this qualitative study may be transferable only to a similar context, bearing in mind that the experience of the women who participated in this study may or may not be identical to the experience of others in a similar situation.

## Conclusion

This study expands the literature on how women react when coincidentally discovering their pregnancy and HIV-positive status during medical consultations. HIV and pregnancy testing are routine services offered by all South African primary healthcare facilities. At that point, the woman receives her result alone without support and undergoes a shock leading to a state of denial and a desire to bargain herself out of the situation. The process of grief varies and women handle the situation differently. Factors such as fear of infecting the unborn baby and emotions arising during this situation can aggravate and cause delay in a woman to accept this situation. However, the transition from denial to acceptance of their pregnancy and HIV-positive status is influenced by various factors such as understanding the HIV infection in general and during the pregnancy and adjustment to live with HIV during pregnancy and beyond that.

Healthcare providers responsible for antenatal and even postnatal care services must handle the situation appropriately by offering the necessary support to these women in need. Support will enable the women to handle the situation as it comes. Communicating the results should be performed appropriately and individual support should be prioritised. Accurate information should be provided to allay the anxiety and worries of women living with HIV during pregnancy. Understanding the implications of living with HIV should be the message communicated to the women when providing psychosocial support to minimise the mixed reaction caused by the negative results a woman was not hoping for. In agreement with other literature recommendations, ongoing counselling and support interventions should be continuously offered to women in every visit to the healthcare facility to facilitate acceptance of the situation and to promote emotional healing. The findings in this study further suggest that there is a need for comprehensive screening for sexual and reproductive health of all women who are still of childbearing age for the early detection of HIV and pregnancy so that women diagnosed with HIV and pregnancy get enrolled on the PMTCT programme at an early stage where counselling will be prolonged. This will help them to cope with the fact that they are going to live life rather differently in future.
